# Influence of Agricultural Land Use Management on Soil Particle Size Distribution and Nutrient Adsorption in Western Kenya

**DOI:** 10.1007/s42250-025-01202-6

**Published:** 2025-03-01

**Authors:** Job Isaboke, Odipo Osano, Olivier S. Humphrey, Sophia M. Dowell, Ruth Njoroge, Michael J. Watts

**Affiliations:** 1 British Geological Survey, Nottingham, UK; 2 University of Eldoret, Eldoret, Kenya

**Keywords:** Soil erosion, Land management, Soil particle size, Macronutrient, Micronutrients

## Abstract

In Kenya, less than 10% of the land is arable, out of which 30% suffers severe soil degradation. This study investigates impact of various agricultural land use management practices (on the concentration and distribution of essential nutrients in different soil particle size fractions (SPSF) within Oroba Valley, Nandi County. Two agricultural plots were selected: Plot 1 (non-terraced) and Plot 2 (terraced cultivation), with topsoil (0–20 cm) sampled using a stratified method. Soil particles were separated by sieving (2000–500 µm, 500–100 µm, 100–50 µm, 50–25 µm) and sedimentation (25–10 µm and < 10 µm). Organic matter (OM), soil pH, and nutrient concentrations were quantified using loss on ignition (LOI), a pH meter in CaCl2, and ICP-QQQ analysis, respectively. Median soil pH was 5.6 for Plot 1 and 5.9 for Plot 2, with OM concentrated at 40% in smaller particles (< 25 µm) and 24% in larger particles (100–2000 µm). Nutrients such as I, Se, Zn, Mg, P, Al, Mn, Fe, Co, Mo, and Cu were concentrated in SPSF of < 10 µm, while Na, K, and Ca were concentrated in medium particle sizes (25 µm–100 µm). Terraced plot (Plot 2) experience greater nutrient loss down the slope compared to Plot 1, that showed nutrient accumulation at lower elevations. Principal component analysis (PCA) revealed that soil pH did not significantly influence element adsorption within SPSFs. Differences in land use management between plots directly impacted nutrient distribution. Optimising agricultural land management can improve soil health, contributing to attaining the United Nations sustainable development goals 1, 2, 6, and 8.

**Supplementary Information:**

The online version contains supplementary material available at 10.1007/s42250-025-01202-6.

## Introduction

Globally, about 80% of arable land experiences severe soil erosion, which is often associated with chronic micronutrient deficiencies (MNDs) that are experienced by > 2 billion people [1–7]. Reduced agricultural productivity, population growth and increasing food demand have led to farmers working on marginal land, often steep slopes > 35 degrees in Kenya, causing further increased soil erosion risk [8–10]. Small-sized soil particles (< 25 µm) are more prone to soil erosion because they have lower mass, less cohesion, higher surface area to volume ratio and less settling tendency, leading to easy movement with erosion agents [11]; as such, an understanding of soil particle size fractions (SPSF) is essential for effective soil management, crop selection, and addressing specific agricultural challenges associated with different soil types; which will lead to, successful and sustainable agriculture on sloped agricultural plots [12, 13].

Soil texture is the proportion of sand, silt, and clay in the soil, and this affects various soil properties, ranging from water retention, drainage, aeration, and nutrient availability [14]. Loam soil, characterised by a balanced mixture of clay, silt, and sand, is often favoured for agriculture due to its optimal water retention, drainage, and nutrient-holding capacity [15]. Since different crops thrive in soils with varied particle size distributions, for example, Saguaro cactus thrives in 0.05–2 mm particle sizes, willows and ferns grow in 0.002–0.05 mm, and rice thrives in clay soils < 0.002 mm; hence knowledge of soil particle size is essential for effective agricultural practices [16]. The SPSF affects the soil's chemical adsorption [17, 18], cation exchange capacity (CEC) [19], soil organic matter content (OM) [20–22] and buffering capacity [23–25]. SPSF directly correlates to the biological properties of soil, such as biomass [26, 27], nitrogen [28, 29], sulphur [30] and carbon cycling [31]. Soil particle size fraction is crucial in crop nutrient bioavailability and bio-accessibility [32]. Different fractions, sand (2000–25 µm), silt (25–8 µm), and clay (< 8 µm), contribute to the soil's overall physical and chemical properties, influencing nutrient availability [33]. Smaller fractions of clay particles have a larger surface area and cation exchange capacity (CEC) than sand and silt, enhancing nutrient adsorption and retention due to their negative charges, with adequate water content essential for nutrient dissolution and transport to plant roots [34, 35]. Soil texture influences root penetration and exploration, and fine-textured soils (clay and silt) can restrict root movement and potentially limit access to nutrients in specific layers [36–38]. Conversely, coarse-textured soils (sand) allow for greater root penetration but may have lower nutrient retention capacity. In addition, sufficient aeration and drainage are crucial for root health and nutrient uptake [39, 40]. Soil structure significantly impacts soil erosion, influencing soil susceptibility to erosional processes [41–43]. Aggregated soils, where plant roots bind particles together, are generally more resistant to erosion than soils with loose structures [44]. Generally, sandy soils characterised by weak aggregation are easily eroded than clay soils that tend to be strongly aggregated [45, 46]. In addition, vegetation cover is crucial in reducing both water and wind erosion as it protects soil surfaces from raindrop impact while reducing wind erosivity [47–49].

In agricultural land, tillage practices affect soil structure and particle size distribution [50]. More tillage (oxen and tractor tillage) increases the breakdown process of large SPSFs to smaller, more erosion-prone particles, whereas zero-tillage or reduced tillage minimises soil disturbance [51–53]. The latter practices help maintain soil structure and reduce erosion [54]. Various cultivation practices are adopted for different regions of agriculture depending on the varied factors, including topography, type of soil, climate, technological advancement and water availability [55]. Furthermore, crop rotation, which entails alternating the types of plants cultivated in a specific area over time, enhances soil quality by optimising nutrient cycling and pest management [56]. The Oroba Valley in Nandi County, Kenya, is characterised by steep hills with an estimated soil erosion loss rate exceeding 20 t ha^−1^ yr^−1^ in some places [49]. Over the last 35 years, farmers have encroached on erosion-prone slopes, employing different tillage practices and adopting various soil erosion control strategies to enhance crop yield and mitigate soil erosion [10]. This encroachment has perturbed the natural soil texture equilibrium, where a balanced soil texture, such as loam soil, is known to correlate with enhanced retention of micronutrients [57–59]. This study aimed to investigate the impact of land management techniques on the concentration and distribution of essential macro- and micronutrients in different soil particle size fractions. The objectives were (1) to determine soil particle distribution of differently used agricultural plots along the slopes of Oroba Valley, (2) to determine the distribution of elements in different soil particle size fractions along the slopes of Oroba Valley, and (3) to investigate the effects of land management on the distribution and adsorption/sorption of macro/micronutrients in agricultural soils along the slopes of erosion-prone areas in Oroba valley.

## Materials and Methods

### Study Area and Sampling Design

**Fig. 1:**
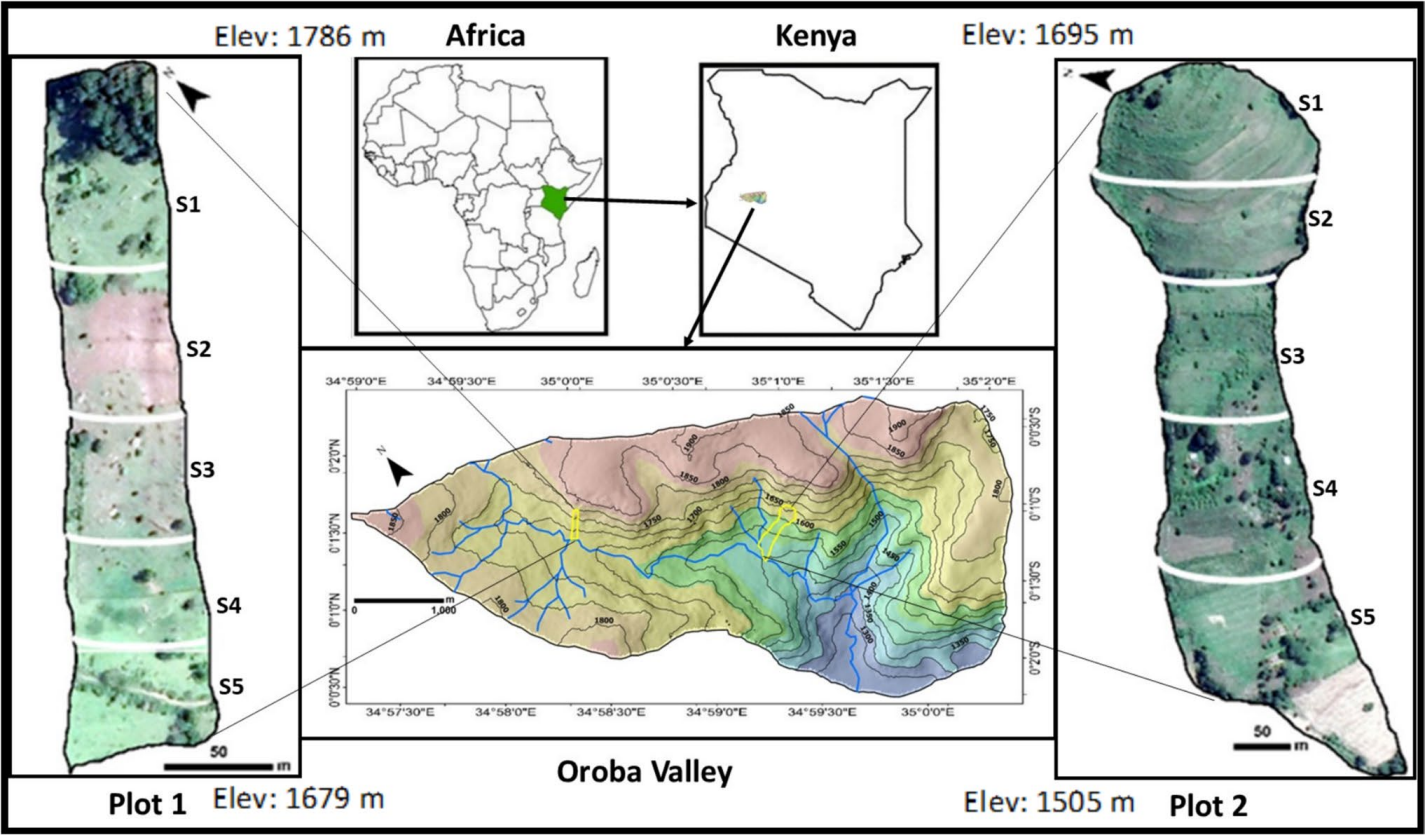
Study plots along River Oroba Valley Winam Gulf, Kenya, and Africa Plot 1- (no-terraces) and Plot 2 (Terraced)

The study was conducted in the Oroba Valley, western Kenya's Nandi hills. The area experiences an equatorial climate, varied rainfall (600≥2000 mm), and temperatures (17.4–29.9 °C); the valley banks towards Lake Victoria with River Oroba passes through the valley and drains into the lake registering high erosion rated of > 20 t ha^−1^ yr^−1^[10, 49]. Two sampling plots on steep slopes in the north-westerly valley were selected: Plot 1, made of Acrisol soil with predominantly practices of conservation farming (no terraces), and Plot 2, characterised by red-brown Cambisol soil with 20-year established terraces predominantly tilled with oxen plough (Fig. 1). Each plot was subdivided into five sections along the slope based on land cover activity and steepness. Three sampling locations were then randomly selected across each section of 1 m × 1 m. At each sampling point, a composite of soil samples was taken using auger flights from three randomly selected points in the 1 m × 1 m area. The auger drilled the sample up to a depth of 20 cm (topsoil). The samples were then dried in the oven at 30 °C until dry before being processed.

### Soil Texture Classification of the Study Site

Dry > 2 mm sieved soil samples were weighed (0.22 g) into a centrifuge tube for particle size analysis, and 10 mL of H_2_O_2_ was added gradually and left for 24 h to react with the organic matter in the soil. The samples were dried in a water bath at 80 °C for 48 h, and then 20 mL of Calgon ((NaPO_3_)_6_ and Na_2_CO_3_) solution was added and shaken for one hour before analysis. Particle size analysis was determined using method described in Rawlins, Wragg and Lark [60] using LS I3 320 Laser Diffraction Particle Size Analyzer. Instrument's resultant output particle sizes ranged from 2000—0.04 µm.

### Soil Particle Size Fractionation by Sieving (2 mm–25 µm).

All samples were dried, sieved to < 2 mm to remove plant remains and stones, and then stored in a dry place. The sieved samples were separated into different-sized portions with 1000, 500, 300, and 212 µm diameters using stacked mesh sieves of respective round aperture diameters mounted on a shaker set for 5 min at an amplitude of 1.5 m. After shaking, all the sieved contents were placed in different labelled sample bags with labels. Contents of the first sieve (stack one portion; < 212 µm) were separated into different-sized portions (100, 50, 25 µm portions) in another set of stacked sieves, shaken for 12 min at an amplitude of 1.5 m and then transferred into sample bags.

### Soil Particle Size Fractionation by Sedimentation According to Stokes Law < 10 µm Fraction

Soil samples sieved to < 25 µm were further separated by sedimentation following Stoke's law. This law states that the force that resists spheres falling in a viscous liquid is directly proportional to the fluids' viscosity and inversely proportional to particle’s diameter. This principle holds true only for spherical particles that are less than 61 µm and under conditions of laminar flow where turbulence is low (Reynolds number R_e_ ≤ 0.2) [61]. In brief, 50 mL of distilled water was added to the 10 g of soil samples in a beaker (beaker 1), and the mixture was allowed to soak on the bench for one hour. The mixture was stirred until all content was thoroughly mixed. The sample was removed from the stirrer plate and allowed to settle for precisely 2 min and 41 s. The supernatant was pipetted and placed in beaker 2. Afterwards, 25 mL of distilled water was added to the remaining contents and stirred for 30 min [61–63]. After the separation process of the soils in the beakers, the content of both beakers (1 and 2) was frozen and freeze-dried at < 40 °C to prevent loss of iodine [64, 65].

### Soil pH and Organic Matter (OM)

Using US EPA SW-846 Test Method 9045D for calcareous soil pH analysis, 12.5 mL of 0.01 M CaCl_2_ was mixed with 5 g of milled SPSFs and calcium chloride slurry (CaCl_2_) to a final ratio of 1:2.5 then stirred and pH measured. Loss-on-ignition (LOI) method was employed to determine (%) organic matter content. After SPSF separation, samples were oven-dried for 24 h at a temperature of 80 °C. One gram of the sample of different SPSFs was ashed in a muffle furnace at a high temperature of 450 °C. Samples were then weighed before and after ashing, and the difference in weight was used to calculate the loss of weight, which was assumed to be due to the loss of organic matter (Singh et al., 2022).

### Soil Nutrients Analysis

Soil samples (whole and size fractioned; 0.25 g) were dissolved on a programmable hot block for digestion in an acid mix of (HF:2.5 mL/HNO_3_:2 mL/H_2_O_2_:2.5 mL) [66, 67]. For iodine, 0.25 g of soil was digested in 5% v/v tetramethyl ammonium hydroxide (TMAH) (5 mL), heated in a Nalgene HDPE bottle at 70 °C for 3 h in a drying oven, and then diluted with 5 mL of deionised water [68], after centrifugation at 3,000 rpm for 15 min, the supernatant was analysed [69]. Soil particle size fraction acid digests were analysed for the total elements carried out by Agilent 8900 ICP-QQQ using collision cell mode (He-gas). Internal standards In, Sc, Rh, Te, Ge, and Ir were employed to correct for signal drift. ICP-MS was utilised in 'no-gas' mode, and separately, all solutions were analysed within a 0.5% TMAH matrix for iodine analysis. Minimal sample utilisation was adopted using micropipettes (Agilent Technologies, USA), and an ISIS-3 sample introduction loop was used to minimise the sample volume (500 µL) presented to the ICP-MS in order to reduce the risk of carryover between samples [68], hence, to avoid inter-sample contamination.

### Quality Control

Various laboratory control measures were implemented to ensure accuracy and reliability of the results. Reference materials utilised in the study were Montana soils (2711a), Basalt (BCR-2), ironstone soils (BGS 102), and soil powder (GSS-2 & GSS-5 for Iodine). These references underwent digestion in duplicate within each dissolving sample batch and were examined thereafter. Laboratory control samples comprising of water with 5% HNO_3_) and 2.5% HCl were analysed to monitor background signals. Additionally, method blanks underwent the same dissolution procedure as the certified reference material (CRM) and samples under analysis. Furthermore, to ensure analytical precision within each batch of dissolution, soil samples were tested three times, thereby confirming consistency and reliability of the analytical data. These laboratory control procedures were implemented to uphold the integrity of the analyses and ensure the results' accuracy. All data used in this paper is in Supplementary Table 1(QC-DATA).

### Statistical Analysis

Excel Office 19, RStudio and SPSS software were used for statistical analyses. Analysis of variance was employed to compare proportional differences and determine significance of the means. Linear regression and correlation were utilised to predict the concentration differences. All analyses employed two-tailed tests, and significance was determined at a p-value of < 0.05 unless specified otherwise. Principal component analysis (PCA) was utilised to assess the impact of soil properties and the co-occurrence of elements. The Enrichment Factor (EF) of nutritional elements is defined as follows, using aluminium (Al) as a reference element:

1 $$\text{EF}=\frac{(Cx/CAl) particle size)}{(Cx/CAl) bulk sample)}$$

where (*Cx*⁄*CAl*) *particle size* is the concentration of the element (mg kg^−1^) and Al (mg kg^−1^) in particle size, and (*Cx*⁄*CAl*) *bulk sample* is the concentration of element (mg kg^−1^) and Al (mg kg^−1^) in bulk soil (< 2 mm fraction) in the study area total topsoil concentration as bulk soil (< 2 mm) [70–73].

## Results and Discussion

### Particle Size Distribution

**Fig. 2:**
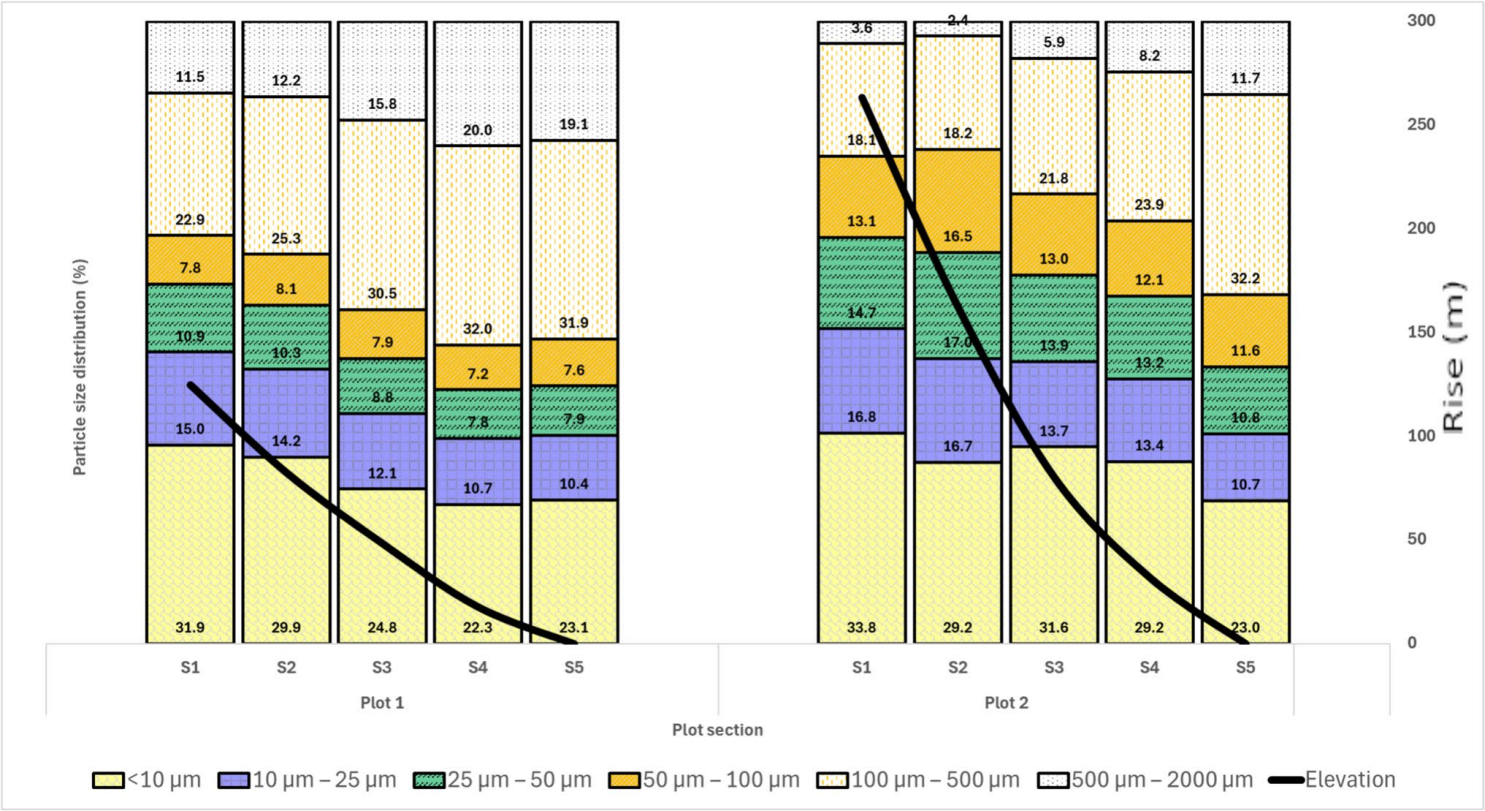
Soil particle size(mm) distribution (%) and Rise (m) within each plot

Measured elevation angle of Plots 1 and 2 slopes were approximately 25.99° and 26.85°, respectively. Soil particle size profile along Plots 1 and 2 generally indicated that the proportion of large soil particles (100 µm—2000 µm) increased down the slope, with a mean percentage increase of 8.4% and 10.8%, respectively. Medium (25 µm–100 µm) and small (< 10 µm–25 µm) particle sizes decrease along the slope of both Plots 1 and 2 with a mean particle percentage of − 4.15% and − 5.58% respectively (Fig. 2).

Soil erosion in the upper part of the Winam Gulf is heavily influenced by slope gradient, slope length, rainfall, and land use [49]. Erosion by water involves velocity of water that leads to the detachment and suspension of soil particles suspended into a runoff. Reduced water velocity leads to decantation of large particle sizes depending on density [74, 75]. Due to this process, large particle sizes are deposited at lower altitudes while water carries light particle sizes into the river streams hence fewer small-size particles in the lower elevation of both studied plots. Reduced tillage farming methods characterise Plot 1, an approach farmers adopt that reduces soil aggregate destruction into smaller particles. On the contrary, Plot 2 predominantly had smaller particle sizes than Plot 1 (Fig. 2), and this was attributed to the breakage of soil particles by the oxen-drawn ploughs/ intense tilling practices.

### Soil pH and Organic matter (OM)

**Fig. 3:**
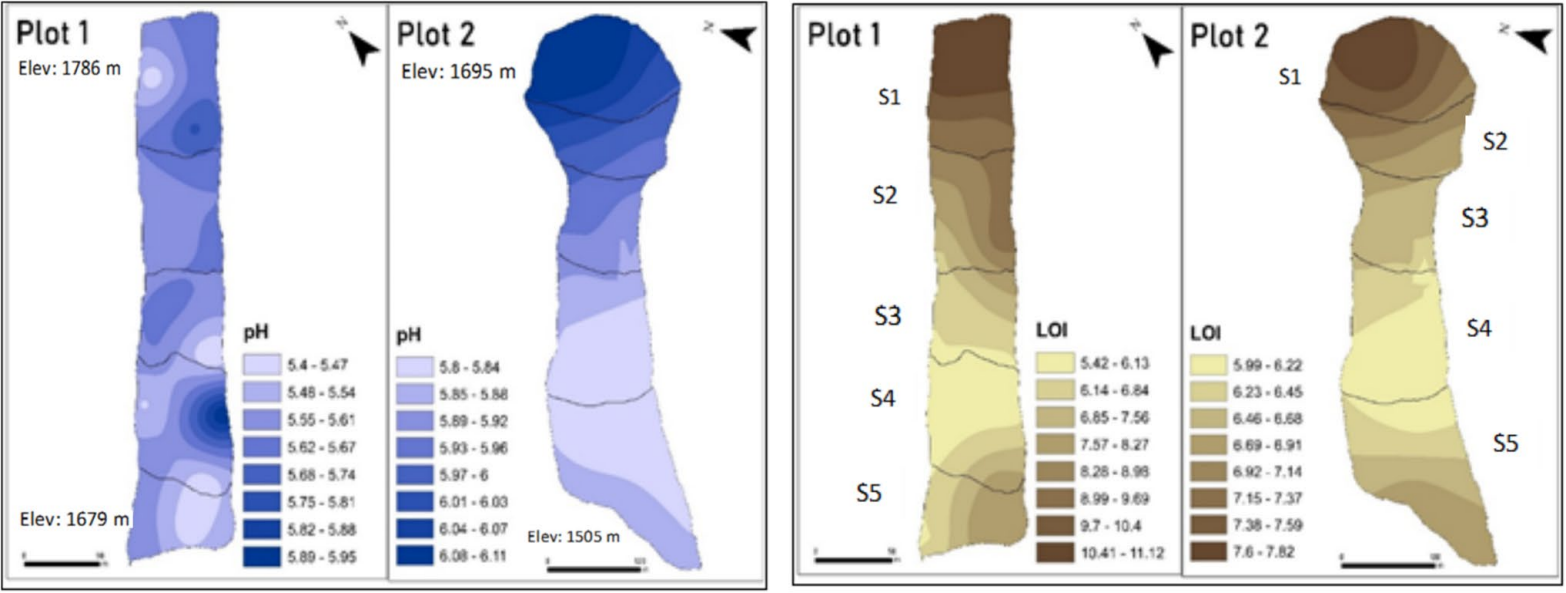
Soil pH and OM distribution maps along the slopes of Plots 1 and 2

Soils from both plots are moderately acidic ranging between, pH of 5.6 and 5.9. Plot 1 has a more even pH distribution compared to Plot 2 which becomes more acidic with decreasing elevation (Fig. 3). Soil pH is an important indicator of the basic physicochemical properties of soil [76–78]. Soil pH influences the binding of elements to soil particles by affecting the ionisation and cation exchange capacity (CEC) of the elements [79, 80]. On Kenya's traditional ecological zone map, these areas are designated as the 'tea-dairy,' 'coffee-tea,' and 'main coffee' climatic zones which have the acidic soils [81]. The acidic pH observed in Plots 1 and 2 could lead to nutrient imbalances and decreases in the availability of vital cations such as Ca, Mg, and K within the study areas soils [19]. In Plot 2, the last two lower sections (S4, S5) host a sugarcane plantation that has remained untouched for the past 10 years, hence impacting the distribution of H^+^ in soil. Root exudation in sugarcane involves the release of various substances from the roots into the surrounding soil, which can significantly influence soil chemistry, nutrient availability, and microbial activity [82]. This acidification enhances the solubility of certain nutrients, such as phosphorus, iron, and manganese, making them more available for plant uptake. Root exudation decreases the soil pH of the surrounding environment, as seen in the results from plots 2, Sects. 4 and 5.

Organic matter (OM) in the study plots generally decreased down the slope. Section 1s of both plots contained the highest quantities of OM ranging from 10.4% to 11.1% and 7.6%–7.8% respectively. The lower elevation of the study plots (Sects. 4 and 5) contains the lowest amount of OM (Fig. 3—OM(LOI)). In the Oroba Valley soils, a positive correlation of r = 0.27, P < 0.01 was observed between pH and OM. Acidity fosters the high accumulation of organic matter, primarily derived from decomposed plant residues [19]. Notably, small particle sizes dominate in both plots, comprising 39% in Plot 1 and 44% in Plot 2, compared to medium and large particle sizes (Fig. 2). The interaction between < 10 µm particle sizes and OM forms clay-humus complexes that stabilise OM, protecting it from decomposition and hence crucial for soil stability and structure enhancement [83–85]. The particle sizes are inversely correlated with OM contents (r = − 0.62, P < 0.01) because the small particle size fractions in soils present higher CEC and adsorption potential due to their relatively larger surface areas [86].

**Fig. 4:**
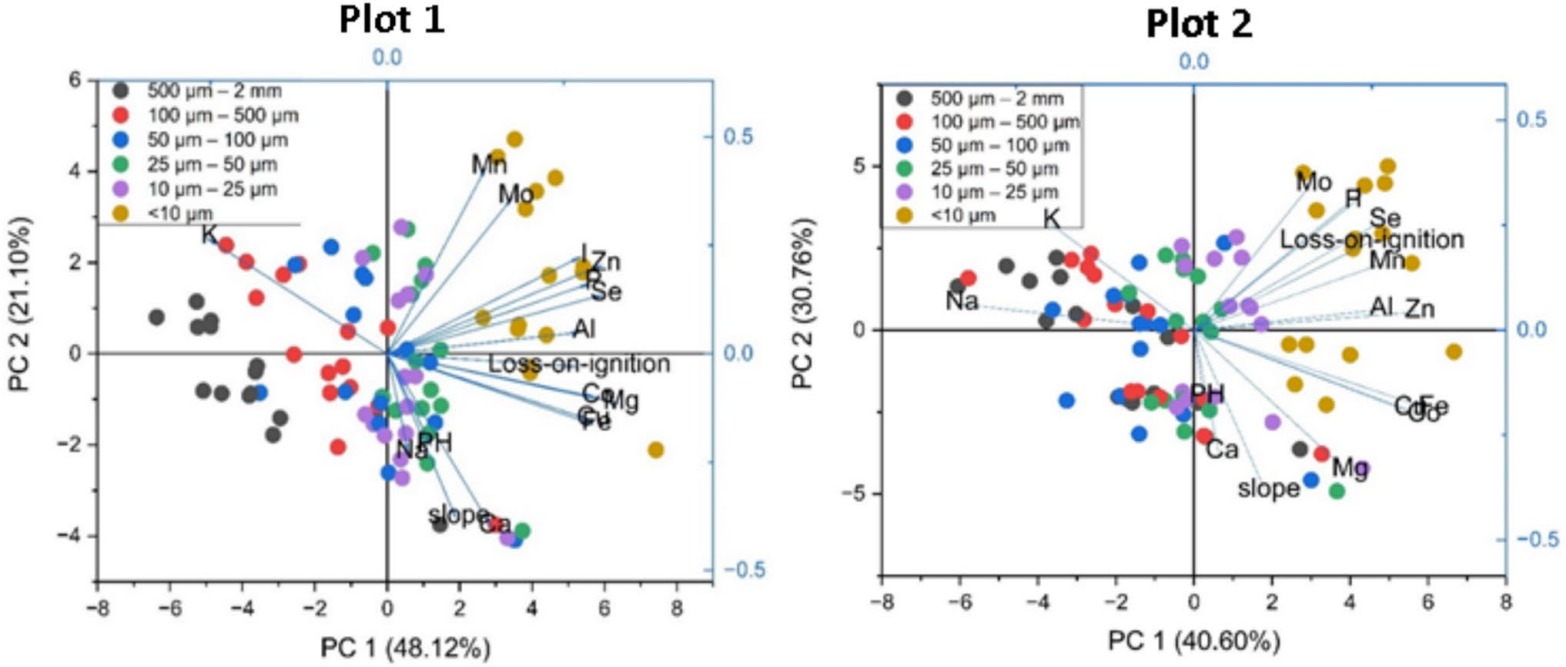
Principal component analysis (PCA) from Plots 1 and 2 for macro-/micronutrients and soil physicochemical properties in different soil particle size fractions. Axis 1 and 2 accounts for 48.12% and 21.10% (Plot 1) and 40.60% and 30.76% (Plot 2) of the variance, respectively

There is a strong correlation between pH and OM, while particle size is a controlling factor for Plot 2 (r = 0.811, P < 0.01) more than Plot 1 (r = 0.243, P < 0.01). Land use activities within each study plot contribute significantly to SPSF formation and the redistribution of OM [87]. For instance, in Plot 1 Sect. 5, designated as a grazing area (Sup—LULC), animal consumption of plant material results in lower organic matter (OM) content. Conversely, in Plot 2, Sects. 3 and 5, being a sugarcane plantation, facilitates more significant plant material decomposition, thus elevating organic matter concentration. The study shows that most of the land uses in the study area lead to OM depletion, as shown in Fig. 4, which correlates with the findings by [88, 89], indicating that land use practices that maintain or enhance plant cover, minimise soil disturbance, and add diverse organic inputs tend to increase OM. Conversely, practices that lead to soil erosion, compaction, and reduced organic inputs typically decrease OM; similarly, tillage is done in Plot 2, Sects. 4 and 5.

### Soil Particle Size Fraction (SPSF) Elemental Distribution

This paper focussed on fourteen macro- and micronutrients, specifically Iodine (I), Selenium (Se), Zinc (Zn), Magnesium (Mg), Phosphorus (P), Aluminium (Al), Manganese (Mn), Iron (Fe), Cobalt (Co), Molybdenum (Mo), Copper (Cu), Sodium (Na), Potassium (K), and Calcium (Ca) In Fig. 4, it is shown that the concentrations of elements were inversely correlated to the soil particle sizes for the soil particles < 10 µm, p-value of < 0.001. However, the pattern was inconsistent with all elements in which Na, K, and Ca displayed the lowest concentrations in the smaller particle size fraction (SPSF) compared to other fractions. The biplots in Fig. 4 reveal the intricate interplay among the physical–chemical attributes of soil, elemental concentrations, and particle size.

Potassium shows a high binding affinity, which is demonstrated by a positive correlation with PC2 and a negative impact on PC1 in Plot 1. In Plot 2, both K and Na exhibit an orthogonal correlation concerning slope and OM (r = − 0.05, P < 0.01, and r = − 0.55, P < 0.01, respectively). Transition of OM from a negative to a positive association with PC2, observed from Plot 1 to Plot 2, unveils the influence of different land management practices. The concentration of the study elements in soils was inversely proportional to the soil particle sizes for small particles (< 10 µm). This finding aligns with the results of Huang, Yuan [46] and Park, Park [90], which demonstrated that nutrient concentrations were elevated in soils with smaller particle sizes (< 2 µm). This concentration is related to surface area, adsorption capacity, and clay minerals, which increase cation exchange capacity [91]. A soil cation exchange capacity (CEC) is a measure of the overall negative charges present in the soil, which attract and bind essential nutrient cations for plants [92], hence dictates the fertility of different soils. Soil pH significantly impacts CEC by influencing the number of negative charges on colloids. As pH increases, the number of negative charges on colloids increases, enhancing their attraction to positive charges. Consequently, this promotes greater binding of elements to the soil particles [93]. Different factors affect the relationship of pH and CEC in elements binding to soil, such as land use and management practices, such as liming, fertilisation, and irrigation, consequently affecting the binding of K and Na to different SPSFs [94]. This is shown in the biplot Fig. 4, where pH has less impact on shaping elemental distribution dynamics.

The analysis of the correlation between pH and the binding of nutritional elements, with SPSF as a controlling factor, indicates that I, Se, Zn, P, Mn, Mo, Na, and K exhibit a negative correlation with pH. This counterintuitive relationship suggests that as pH levels increase, the concentrations of these elements tend to decrease. The remaining elements, Mg, Al, Fe, Co, Ca, and Cu, indicated a positive correlation with pH, indicating a direct association between pH levels and their abundance in the soil (Supplementary Table 2—correlation)*.*

There is a positive correlation between OM% and elemental concentrations across all SPSF which indicates that an increase in organic matter content leads to increased concentrations of elements within the soil matrix (Fig. 4; Supplementary Table 1). This correlation holds true for most elements across both plots, except for Na, K, Mg, Mn, Fe, and Ca. In Plot 1, where NPK fertiliser is employed during planting, elevated quantities of Na and K are observed (Sup- Figs. 7 and 9, maps and distribution), with mean concentrations of 17,358 mg kg^−1^ and 25,406 mg kg^−1^, respectively, compared to Plot 2, where mean concentrations of Na and K, were 11,175 mg kg^−1^ and 14,880 mg kg^−1^, respectively. This variation was attributed to the differences in land management, in which the no-minimal tillage practices adopted in Plot 1 (non-terraced) mitigate erosion and facilitate the accumulation of elements within smaller particle sizes (< 10 µm). Elevated amounts of element concentrations in small SPSF result from the decomposition and transformation of plant and animal tissues by edaphons, as indicated by OM % measured [95].

Interestingly, Na, K, and Ca exhibit a negative correlation in Plot 2, indicating their preferential concentration in 500 µm–2000 µm SPSFs. Specifically, Na is found to concentrate in the 500–100 µm size fraction in Plot 2, with a mean concentration of 18,084 mg kg^−1^, suggesting its propensity to disperse and exhibit reduced attraction to smaller SPSF due to ion size dynamics [96]. Larger soil particles (25 µm–100 µm) were sodic (concentration of Na, 991,872 mg kg^−1^) in concordance with Rengasamy [97] and Rengasamy and Olsson [98].

**Fig. 5:**
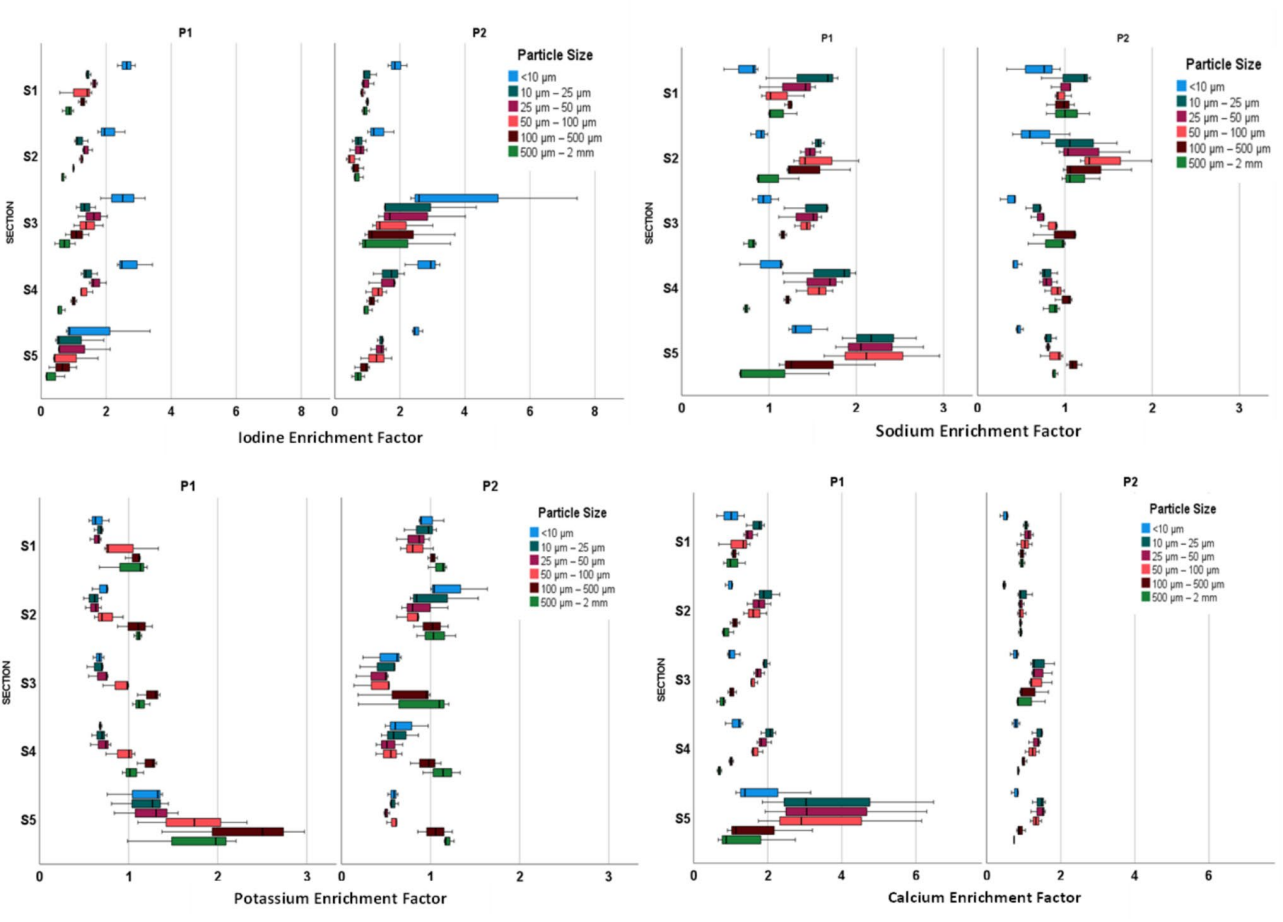
Enrichment factor distribution of Iodine (I), Sodium (Na), Calcium (Ca) and Potassium (K) elements across the slopes of Plots 1 and 2 in different soil particle sizes

Figure 5 illustrates the distribution data of Na, Ca, K, and I along the slopes of the two study plots. In Plot 2, Ca concentrations notably exceeded that of Plot 1, averaging 16,841 mg kg^−1^ and 12,530 mg kg^−1^, respectively (Fig. 5). The distribution of Ca across particle sizes was consistent, showing a marked preference for particle sizes between 10 and 25 µm with a median of 17,103 mg kg^−1^. This behaviour is attributed to Ca being a divalent cation that interacts with anions, facilitating the aggregation of particles [99, 100]. The study plots exhibit aggregation of soil particle sizes rich in Ca, and all medium SPSF have relatively higher concentrations, with a mean of 17,978 mg kg^−1^.

On average, the amount of K concentration in larger size fractions (> 100 µm) was statistically significantly (p-value < 0.001) higher than the other fractions measured in all the study plots (Fig. 5). This finding aligns with studies on K fixation, which show that the sand fraction exhibits a larger capacity for K fixation compared to silt and clay fractions [101].

Results indicate that K in 100 µm–500 µm contained a concentration of 33,666 mg kg^−1^ across both study plots along the slope. In Plot 1, K showed higher concentrations with a median of 29,176 mg kg^−1^ at lower elevations, suggesting it had been washed down the slope. According to Fig. 5, Plot 2 exhibited the lowest concentration of K compared to Plot 1. Soil composition also plays a crucial role in the binding of micro- and macronutrients to soil particle sizes. Micas, Hydrous Micas, or Vermiculites in the soil increase the binding of K to soil more than soil with smectite and kaolinitic have low fixation capacity [102]. Moreover, farmers in western Kenya commonly use NPK fertilisers during planting [103, 104], enriching the soil with significant K levels to enhance plant physiology. Large SPSFs (> 100 µm) of soils in Oroba Valley contain hydroxyl-interlayered vermiculite, whereas small SPSFs contain smectite [105]. Due to soil characteristics, smectite releases K for plant availability, whereas vermiculite fixes it to soil and is unavailable for plant uptake [106]. Smaller soil particles, < 10 µm, often boast a larger surface area: volume ratio and exhibit higher surface charge, leading to enhanced adsorption of cations like I, Se, Zn, Mg, P, Al, Mn, Fe, Co, Mo, and Cu in our study.

Generally, the concentrations of I, Se, Zn, Mg, P, Al, Mn, Fe, Co, Mo, and Cu decrease with increasing soil particle size, reflecting the higher CEC of smaller SPSFs compared to larger ones, such as silt and sand [107]. Moreover, the specific mineral composition of the clay fraction can also influence the adsorption capacity [108]. In some cases, as shown by Mg in this study, certain minerals in larger particle sizes (> 100 µm) may have a relatively higher affinity for cations [109]. The correlation between the SPSF elemental concentration differed from one element to another. Magnesium displayed no significant variation between SPSFs within Plot 1 but exhibited a significant difference in Plot 2 (p-value < 0.001). Conversely, all other elements analysed exhibited significant differences between SPSFs in both plots (p-value < 0.001). Land management practices at the study site play a vital role in modelling the formation and distribution of SPSFs, thereby influencing changes in concentration variance among SPSFs in different plots (Fig. 2). In particular, micronutrient concentrations decrease with increasing soil depth [110]. Farmers in Plot 2 have implemented terrace cultivation to mitigate soil loss along slopes, resulting in the transfer of soil from higher to lower ground and the formation of more fine particles in Sects. 1 and 2. This redistribution process has contributed to the depletion of elements on higher ground, consequently impacting the elemental concentration distribution across SPSFs within the plots.

Plot 1 adopted conservation farming practices, resulting in minimal disturbance to the land area, and consequently, fine particles are less generated. Soil particle size fractions redistribution by water movement also accumulates elements in 2000–1000 µm size fraction [111]. The study area occurs in an erosion-prone region highly susceptible to year-round heavy rainfall [112]. Small and medium-sized particles are transported in Plot 2, where the water velocity is reduced by overgrown sugarcane plantations (S4, S5). This decants water, leading to soil deposition, hence the bioaccumulation of elements in Sect. 5 [10, 49]. Farmers graze cattle in both study plots between the crop-growing seasons. Farmers in the study plots supplement cattle feed by adding salts, which are sprinkled directly into the cow feed. Also, using diammonium phosphate (DAP) and NPK fertilisers in the planting period increases the amount of K and P in the soil. The addition of Na, K, and Ca are likely to be redistributed into surface runoff via heavy rains, particularly during periods of reduced ground cover [49].

## Conclusions

The study investigated the impact of different land use management scenarios on the concentration of macro and micronutrients in different particle sizes. From the study, PCA indicated that pH least affected the distribution of elements in different SPSFs along the slopes. Farmers of the study plots leave the crop residues on the farm after harvesting, increasing OM in the soil, which is an essential factor influencing elemental binding to small particle sizes in the study area. Land use management in relation to erosion factors influences the dispersion of elements across slopes and within the SPSF of the study plots. Conservation farming in Plot 1; No or low tillage is preventing breakages of soil into small particles hence more concentrated as most elements are in smaller particle sizes, hence more easily transported down slopes through surface runoff. In Plot 2, the physical movement of soil redistributed the elements in soil particles, but plantations and continuous land cover in lower elevations (S4, S5) reduced erosion and therefore the overall loss of elements. Maintaining land cover along the slopes of steep hills and reduced tillage cultivation reduces the amount of small particles generated; this, in turn, reduces the concentration of elements lost either by water or wind, as justified by the majority of elements being accumulated in the small particle size (< 10 µm). Optimising land management practices such as crop rotation, mulching, and conservation tillage enhances soil fertility and crop yields, which will be important factors contributing to food security (SDG 1 and 2). Additionally, adopting soil conservation measures and diversifying crop production can increase farmers' income and nutritional diversity and improve environmental resilience (SDGs 6 and 8).

## Supplementary Information

Below is the link to the electronic supplementary material.

Supplementary file1 (XLSX 4141 KB)

## Data Availability

Data will be made available on request.
